# Reliability and Validity of Mini-Balance Evaluation System Test in Type 2 Diabetic Patients with Peripheral Neuropathy

**DOI:** 10.3390/ijerph19116944

**Published:** 2022-06-06

**Authors:** Sitt Nyein Phyu, Punnee Peungsuwan, Rungthip Puntumetakul, Uraiwan Chatchawan

**Affiliations:** 1 Faculty of Graduate School, Khon Kean University, Khon Kaen 40002, Thailand; sittnyein@kkumail.com; 2 Research Center in Back, Neck, Other Joint Pain and Human Performance (BNOJPH), Khon Kean University, Khon Kaen 40002, Thailand; ppunne@kku.ac.th (P.P.); rungthip@kku.ac.th (R.P.); 3 School of Physical Therapy, Faculty of Associated Medical Sciences, Khon Kaen University, Khon Kaen 40002, Thailand

**Keywords:** balance, diabetic peripheral neuropathy, Mini-BESTest

## Abstract

Type 2 diabetic peripheral neuropathy is known to cause balance limitations in static, dynamic, and functional activity. The Mini-BESTest, a shortened version of BESTest, was evolved to identify balance disorders within a short duration. No prior studies have yet been conducted to assess the usefulness of Mini-BESTest in the diagnosis of type 2 diabetic peripheral neuropathy. The current study aimed to examine the reliability and discriminant validity by comparing the Mini-BESTest scores between type 2 diabetic patients with peripheral neuropathy, divided into two 2 groups based on reporting scores of <4 and ≥4 in the MNSI questionnaire, respectively. Therefore, a cross-sectional study design was conducted including 44 type 2 diabetic patients (4 males and 40 females; aged 56.61 ± 7.7 years old). Diabetic peripheral neuropathy was diagnosed by physical assessment using the Michigan Neuropathy Screening Instrument (MNSI). Inter-rater (two physiotherapists) and Intra-rater (7–10 days) reliability of the Mini-BESTest were explored with intraclass correlation coefficients (ICC_2,1_) and (ICC_3,1_). The Mini-BESTest presented an excellent inter-rater reliability (ICC_2,1_= 0.95, 95% CI = 0.91–0.97, SEM = 0.61) and an excellent intra-rater reliability (ICC_3,1_ = 0.93, 95% CI = 0.87–0.96, SEM = 0.66), with confirmation by a good agreement presented by the Bland–Altman plots. The internal consistency measured with the overall Cronbach’s alpha showed an acceptable agreement (0.73). The MDC was 2.16. In addition, the Mini-BESTest scores in the type 2 diabetic neuropathy patients reporting MNSI questionnaire scores <4 was found to be significantly higher when compared with those reporting scores ≥4. The Mini-BESTest can be used as a highly reliable and valid clinical application in the population with type 2 diabetic peripheral neuropathy.

## 1. Introduction

According to the International Diabetes Federation (IDF) [1], the prevalence of diabetes in Myanmar has recently been estimated as 3.9% in adults aged 20–79 years of age, out of which 90% have been diagnosed with type 2 diabetes [2]. Moreover, half of the total diabetic population suffers from diabetic peripheral neuropathy (DPN), a chronic complication affecting the nerves which starts with peripheral sensory nerve damage, followed by progressive disorders of the motor and autonomic nerves [3]. The prevalence of DPN was estimated at 33.7% in Yangon, Myanmar according to the out-patient clinic data of four hospitals in Yangon. In addition, older age and prolonged diabetic duration were identified as risk factors for the development of DPN [4].

Balance or postural control is a process of complex interactions involving sensory, motor, cognitive, and cardiovascular control [5]. Thirty percent of DPN patients present reduced ankle reflexes, muscle weakness, loss of balance, coordination, and gait stability [6,7], while balance impairments in static, dynamic, and functional stability are also commonly found [8,9]. The impaired sensory function causes a decline in proprioception, movement strategy, cognitive performance, and balance performance as well as the occurrence of biomechanical structural disorders and disorientation [10,11]. Hence, DPN severely affects patients’ ability to walk and perform various other daily activities and promotes the incidence of fall-related injury [12,13,14].

Identification of balance impairments associated with these disorders in type 2 diabetes with or without peripheral neuropathy is typically done with high technology machinery or with clinical balance measures which do not examine all balance control systems. Therefore, simpler, and non-invasive validated tools for multi-system balance measurement are urgently needed.

Previous studies reported the use of balance measure tools such as the Berg balance scale (BBS) and Performance-oriented mobility assessment (POMA); however, several issues limiting their clinical applicability were identified. For example, Jernigan et al. (2012) presented the validation of balance measures in distinguishing between fallers and non-fallers in the DPN population and reported that both BBS and POMA were used in the assessment of somatosensory and visual systems displayed ceiling effects [15].

The Balance Evaluation System Test (BESTest), originally developed for persons with Parkinson’s disease [16], covers all balance control systems affected in the diabetic population [17]. The Balance Evaluation System Test (BESTest) includes six subscales: (1) biomechanical constraints, (2) limit of stability and verticality, (3) anticipatory postural adjustments, (4) postural responses to external perturbations, (5) sensory orientation during stance, and (6) gait stability. Since the total duration of administering the BESTest is approximately 30–45 min, it was determined that the long period of administration may limit its usefulness in a clinical setting [16]. Consequently, an abbreviated version of BESTest eliminating redundant items was proposed in order to allow administration in only 10–15 min. The resulting test is referred to as the Mini-Balance Evaluation System Test (Mini-BESTest).

The Mini-BESTest includes only four sections: (1) transitions/anticipatory postural control, (2) reactive postural control, (3) sensory orientation, and (4) stability in gait [18]. Due to allowing the examination of almost all components of balance control systems (static and dynamic stability, basic motor control systems, stability limits, reactive and anticipatory controls, sensory integration, and cognitive influences) [19] within a short duration, the Mini-BESTest is deemed more useful in a clinical setting than other balance measures. Moreover, the Mini-BESTest is focused specifically on dynamic balance control, including the capability to react to postural perturbations, stand on a compliant or inclined surface, or walk while performing a cognitive task, which represents a significant advantage over other popular balance scales such as BBS [18]. All these features of balance control are known to be important in clinical use for assessing balance disorders in different types of patients and reflect balance challenges during daily living activities [16,20].

The Mini-BESTest provides rating categories with high reliability and structural validity [18] and Marques et al. (2017) concluded that the BESTest and Mini-BESTest are suitable tools to assess the fall risk in older diabetes persons, showing good concurrent validity with Spearman’s correlation coefficients ranging from 0.85 to 0.91 (*p* < 0.001) [21]. However, since this represents the only comparative study conducted thus far, other forms of validity tests, such as construct validity, may also need to be evaluated in order to confirm general validity.

Based on a psychometric review, the Mini-BESTest has also been shown as a standardized balance measure with reliable and valid test performance in other neurological and musculoskeletal diseases, such as stroke, Parkinson’s disease, knee arthroplasty, etc., but has not been studied in the DPN population to date.

The Mini-BESTest has not yet been widely used in clinical practice and, despite the advantages mentioned above, studies investigating whether the Mini-BESTest may be useful in type 2 diabetic populations diagnosed with peripheral neuropathy have not been reported to date. Therefore, the purpose of this study was to explore the reliability and validity of the Mini-BESTest as a short and quickly assessable tool in type 2 diabetic patients with peripheral neuropathy.

## 2. Materials and Method

### 2.1. Study Design

A cross-sectional study was conducted on type 2 diabetic patients with peripheral neuropathy who were recruited from an outpatient diabetic clinic at North Okkalapa General Hospital, Yangon, Myanmar. The study protocol followed the guidelines of the Declaration of Helsinki and was approved by the Human Ethics Committee of Khon Kaen University, Thailand (protocol number: HE632104) and the Institutional Review Board (IRB) of the University of Public Health, Myanmar. Each participant was provided a written statement of informed consent prior to participating in the study and was requested to sign the consent form before data collection.

### 2.2. Participants

**Figure 1:**
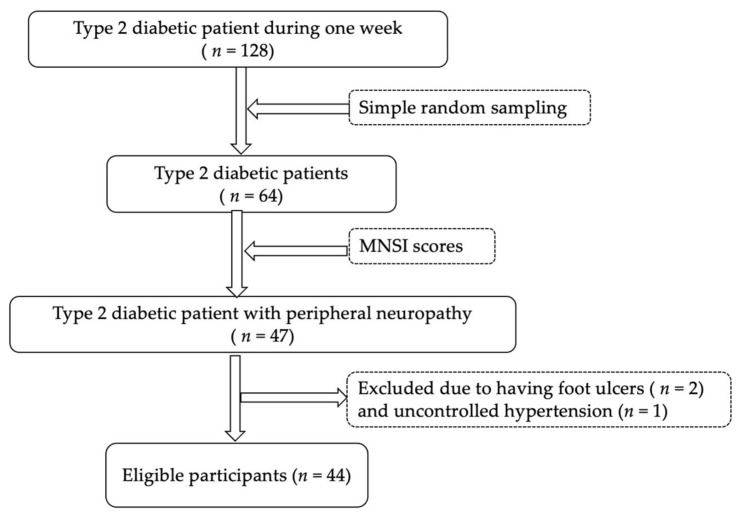
The flow diagram of the participant recruitment process.

A total of 128 patients diagnosed with diabetes who were visiting the hospital during one week (research recruitment period) were approached as the target population. Out of these, 64 participants were selected using a simple random sampling method (using hospital numbers). In total, 47 participants were diagnosed with having DPN and 3 participants were excluded; therefore, 44 male and female type 2 diabetic patients with peripheral neuropathy, aged 40–70, were found eligible for our study and remained in the trial group (Figure 1). Each patient was screened using the Michigan Neuropathy Screening Instrument (MNSI), including both subjective (15 questions) and physical (10 scores) examinations. Patients’ subjective examination scores were classified to be less versus more severity of DPN (<4 vs. ≥4). A cut-off point of 2.5 out of 10 scores of physical examination (assessment of present or decrease or absent) was used to confirm the diagnosis of DM with DPN in participants who had a physical assessment score from the Michigan Neuropathy Screening Instrument (MNSI) [22]. The inclusion criteria were as follows: (1) more than 5 years of diabetic duration, (2) well-controlled blood glucose based on three follow-up medical assessments, and (3) ability to understand the instructions as judged by mini-mental state examination (MMSE) [11]. Exclusion criteria were as follows: (1) having foot ulcers and/or fracture of lower limbs within six months before the study, (2) having peripheral venous insufficiency, cardiac, renal, or hepatic insufficiency, uncontrolled hypertension or myopathy, central nervous system dysfunction, or partial or complete blindness, and (3) severe auditory problems as evidenced by medical record or physical examination [21,23].

### 2.3. Procedures

**Figure 2:**
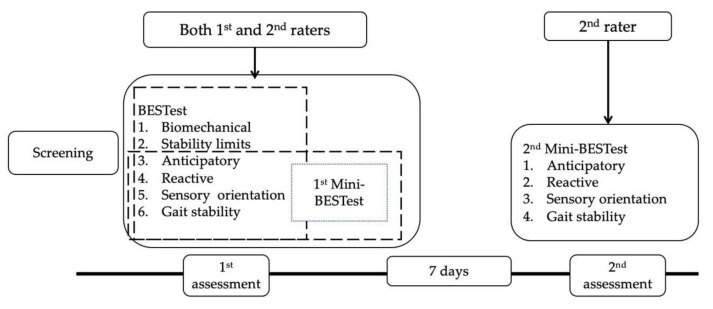
Study procedure.

Figure 2 displays the general study procedure. Each patient was instructed to receive testing for 2 days in a quiet laboratory room in a thermoneutral environment of 25–27 °C. On the 1st day, the demographic characteristics and medical record (level of blood glucose and HbA1C) of each participant was recorded and the patients were screened for DPN by a research assistant (a physical therapist with 5 years of experience). Thereafter, each patient was observed and scored independently and concurrently by two raters for the 36 items of the BESTest, including 28 items of the Mini-BESTest, while the research assistant gave the instructions to the patients. The second assessment for the 28 items of the Mini-Bestest was done after 7 days and was scored only by the second rater.

Both raters were clinical physical therapists with 15 and 8 years of experience, respectively. Prior to the trial, the research assistants and both raters studied training videos including instructions on how to perform and score the test, taken from the official website of the BESTest scale (www.bestest.us accessed on 12 February 2020).

### 2.4. Sample Size

The sample size was estimated based on the intraclass correlation coefficient (ICC) using STATA version 12 and calculated using an ICC of 0.92 in the Mini-BESTest [24] as the value of the Hypothesis value (p1) and using ICC = 0.80 as the Null value (p0) with two repeated measurements and using 10% for a loss of follow-up. As a result, 44 participants were required.

### 2.5. Material and Assessment Tools

#### 2.5.1. Balance Evaluation System Test (BESTest)

The BESTest is composed of 27 different tasks evaluating six subsystems of balance control: (1) biomechanical constraints, (2) stability limits/verticality, (3) anticipatory postural adjustments, (4) postural response to external perturbations, (5) sensory orientation, and (6) stability in gait. Since some items are assessed for both the right and left sides of the body, the total number of items is 36. Each item is scored on a 4-point ordinal scale ranging from 0 (severe balance impairment) to 3 (no balance impairment), adding up to a total score of 108 with higher scores indicating better balance [16]. BESTest was proven to have a good convergent validity (rho from 0.62 to 0.70; *p* < 0.001) with Activities-specific Balance Confidence (ABC) in older adults with type 2 DM to identify fall risk [21].

#### 2.5.2. Mini-Balance Evaluation System Test (Mini-BESTest)

The Mini-BESTest, the shortened form of BESTest, contains only 14 different tasks and involves four sub-sections: (1) anticipatory postural adjustments, (2) postural response to external perturbations, (3) sensory orientation, and (4) stability in gait [18]. Two items of Mini-BESTest are assessed bilaterally, but only the lower score was added to the maximum score of 28 points, which results from grading based on a 3-point ordinal scale ranging from 0 (severe balance impairment) to 2 (no balance impairment) [25].

### 2.6. Data Analysis

Descriptive statistics were used for the demographic data of the participants. The reliability of the Mini-BESTest was analyzed for both categories (ordinal scales of each item) and continuous data (total scores).

For the ordinal scales of each item of the Mini-BESTest, measurements by the first rater (expert) were used for testing the internal consistency, which included Cronbach’s alpha coefficient and the corrected item-total correlation. A Cronbach’s alpha >0.70 [26], and item-total correlation values >0.20 [27] were considered as satisfactory. Single item agreement of the Mini-BESTest score between two raters and between two assessments (second rater) was calculated by using the Weighted Kappa coefficient [26]. For the continuous data of the Mini-BESTest score, these data measurements were examined using the Shapiro–Wilks test before the reliability test.

The inter and intra-rater reliability were analyzed using the intra-class correlation coefficient (ICC) with model 2,1 (two-way random-effects or ICC_2,1_) and model 3,1 (two-way mixed effect or ICC_3,1_), respectively. For the inter-rater reliability, 14 tasks of the Mini-BESTest scored by the first rater (expert) and second rater were analyzed. For determination of the intra-rater reliability, the data for the 14 tasks of the Mini-BESTest scored by the second rater on days one and two were used.

Both inter/intra rater reliability tests were confirmed by Bland–Altman (B&A) plots for the graphical representation of differences between raters (first vs. second rater) and between two assessments from the second rater. The Bland–Altman analysis was also used to determine the difference between the two measurements against the mean scores for each subject, as well as to identify the bias (mean difference) of the measurements and the 95% limits of agreement (LoA) [28,29]. In addition, a linear regression with the coefficient of determination was performed.

The standard error of measurement (SEM) was calculated based on the result of ICC from both inter and intra-rater reliability and the minimum detectable change (MDC) was established on the result of multiplication of the SEM × z value × √2 [30]. MDC was calculated using the data from the second rater.

Construct validity was evaluated using the discriminative validity (which constitutes a type of construct validity) [31] using an independent t-test to determine the difference of the Mini-BESTest and BESTest scores between the participants whose reported MNSI questionnaires (subjective examination) were ≥4 versus those who reported <4 [22]. The 95% confidence intervals (CI) of each assessment were presented and the respective *p*-values were used to determine the level of significance.

## 3. Results

The demographic characteristics of the participants are compiled in Table 1. Overall, Cronbach’s alpha of reliability statistics for all items was 0.73, indicating acceptable agreement. Almost all items of the Mini-BESTest showed satisfactory item-total correlations ranging from 0.31 to 0.52, except for item 1 (sit to stand), item 7 (stand on a firm surface), and item 12 (walk with pivot turns) in Table 2. Weighted kappa values for each item (excluding items 1 and 7) presented moderate to perfect single item agreement. The weighted kappa value between raters ranged from the lowest value of 0.6 for item 4 (compensatory stepping correlation-forward) to the highest value of 1.00 for item 12 (walk with pivot turns). Weighted kappa values for the test-retest agreement coefficient ranged between 0.59 for item 4 (compensatory stepping correlation-forward) and 1.00 for item 12 (walk with pivot turns). There was no elicit given for item 1 (sit to stand) and item 7 (stand on a firm surface) because the same scores resulted from the raters for inter-rater reliability and intra-rater reliability (Table 2).

Table 3 shows the inter-rater reliability displayed high reliability with an ICC = 0.95 (95% CI = 0.91 to 0.97, *p* < 0.001, SEM = 0.61), whereas an ICC = 0.93 (95% CI = 0.87 to 0.96, *p* < 0.001, SEM = 0.66) was found for intra-rater reliability. The MDC95 of the Mini-BESTest was 2.16.

Table 4 reveals the mean difference of Mini-BESTest scores between the participants reporting MNSI questionnaire scores ≥4 and <4 was 2.09 (95% CI = 0.52 to 3.66 ± 0.76, *p* = 0.0102) while the mean difference of BESTest between the two groups of the participants was 4.40 (95% CI = −0.41 to 9.20, *p* = 0.0718).

**Figure 3:**
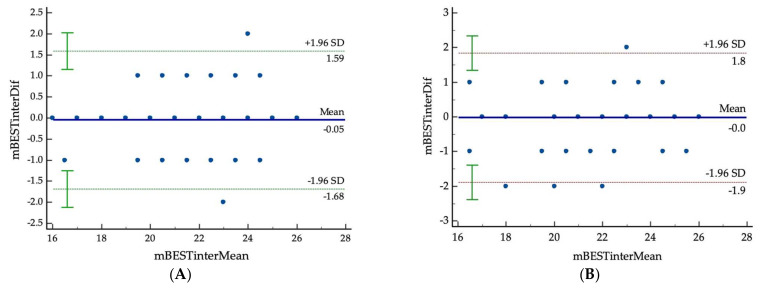
The Bland–Altman analysis of graphical representation of differences between raters (first vs. second rater) (**A**) and differences between two assessments from the second rater (**B**). The solid line in the middle represents the mean difference value of the sample (*n* = 44) due to the same value (26 dots are present in the graph). The dashed lines represent the upper and lower limits of agreement between the two data sets (mean ± 1.96 SD). mBESTinterDif = The difference of the Mini-BESTest total score; mBESTinterMean = The mean of the Mini-BESTest total score.

A Bland–Altman (B&A) analysis was performed using the scatter diagram of the differences plotted against the averages of the two measurements. Horizontal lines were drawn at the mean difference and at the limits of agreement which were defined as the mean difference ± 1.96 SD of differences. Figure 3A shows the total score between the two raters (the first vs. second rater). Almost all data (*n* = 42 or 95.5%) were within the limits of agreement. The mean difference of the Mini-BESTest total score between the two raters was −0.04545 ± 0.834 which presented a distribution of the difference within the limits of agreement and without a statistically significant difference from 0 (*p* = 0.7195). The regression model was defined as:

*The Difference of the Mini-BESTest= −1*.*62+0*.*07 mean Mini-BESTest* (with *p* = 0.140 and R^2^ = 0.0512).

Similar results were found for graphical representation of the difference between two assessments from the second rater (Figure 3B). Almost all data (*n* = 40 or 90.95%) were within the limits of agreement. The mean difference of the Mini-BESTest between the two raters was −0.023 ± 0.952 which presented a distribution of the difference within the limits of agreement and without a statistically significant difference from 0 (*p* = 0.8749). The regression model was defined as:

*The Difference of the Mini-BESTest= −0*.*61+0*.*03 mean of Mini-BESTest* (with *p* = 0.652 and R^2^ = 0.0049).

From the results above, both the data from the two raters or two measurements from the same rater were considered to be in agreement and may be used interchangeably.

## 4. Discussion

The present work constitutes the first study to investigate the reliability of the Mini-BESTest in type 2 diabetic patients with peripheral neuropathy. The Mini-BESTest showed excellent results in terms of reliability and exhibited a strong correlation with the BESTest in the studied population.

The internal consistency was found to be satisfactory, although lower than in other studies (Cronbach’s alpha ranging from 0.89 to 0.96) [30,32,33,34]. The item to total correlation was found to have the lowest value for item 1 (sit to stand), item 7 (stand on a firm surface), and item 12 (walk with pivot turns).

The weighted kappa between the two raters and between two assessments are found to be in moderate to perfect agreement, highly similar to the perfect agreement finding in a recent study for the subacute stroke population. The difference was a moderate coefficient found in item 14 (dual-task training) in test-retest and item to total correlation in that study. In the present study, the lowest agreement is found in item 4 (compensatory stepping correlation-forward) between raters and between two assessments. Tsang et al., 2013, explained that for the compensatory stepping tests, the lowest agreement in scores might be related to the consistency of the therapist applying the displacement. The patients’ balance response may be different according to a slight increase or decrease in the magnitude of the displacing force applied by the therapist [34]. The lower agreement in different items in this work may be a result of the different study population and the younger age group than in the preceding study. Another reason may be that the diabetic population with peripheral neuropathy achieved better balance performance than populations with other neurological diseases (i.e., the subacute stroke population or Parkinson’s disease population). Hence, nearly all participants received higher and largely similar scores from both raters and in both assessments.

The weighted kappa statistics for single item agreement have not been elicited for item 1 (sit to stand) and item 7 (stand on a firm surface), which may be due to the fact that similarly high scores were given by both raters at both times for all participants. This explanation is consistent with the results of a previous study in which almost perfect scores in these items from all raters were reported, as well as with the finding that these results were likely to be influenced by the ease of the task [35].

Single item agreement could not be elicited for item 1 (sit to stand) and item 7 (stand on a firm surface) which may have caused the single to total item agreement score to be the lowest in these items. Moreover, item 12 (walks with pivot turns) having the highest single item agreement showed a lower item to total correlation agreement than other items. A similar observation was reported in a previous study by Lampropoulou et al., 2019, who found that the highest single agreement item had elicited the lowest item to total correlation [33].

The Mini-BESTest can be assumed to provide a reliable tool in the diabetic population to give stable and consistent results over time and between raters for each item and total scores. This may be proven by a variety of results, such as the moderate agreement of individual items within two raters and within two assessments and good internal consistency of Mini-BESTest’s items. Supporting this assessment, excellent reliability of the Mini-BESTest total score when assessed with Intraclass Correlation Coefficient (ICC) between raters and between repeated assessments was also proven.

The inter-rater reliability assessed the correlation between the scores given by two raters in real-time instead of video taking. The high inter-rater reliability of the current study is in accordance with previous reports concerning the application of Mini-BESTest to other populations. For example, excellent ICCs of inter-rater reliability between 0.91 to 0.99 were documented for chronic stroke patients [33], older adults living in nursing homes [36], and patients with balance disorders of different origins [30] or Parkinson’s disease [24].

The intra-rater reliability was used to determine the relationship between two assessments performed under identical conditions within 7 to 10 days, in analogy to protocols defined in earlier studies [30,33,36]. The results obtained in the present work are found to be similar to reported test-retest reliabilities between 0.80 and 0.98 within the population of chronic stroke [33], balance disorders [30], Parkinson’s disease [24], and older adults [36].

The excellent inter/intra reliability tests of Mini-BESTest were confirmed by the results of the Bland–Altman analysis, which presented a good agreement in the measurements between raters and between two assessments of the second rater. These findings are similar to the results of previous studies on Parkinson’s disease and stroke populations [33,37,38].

The Mini-BESTest is, therefore, deemed a valuable and reliable tool for the diabetic population, giving stable and consistent results over time and between raters for each item and total scores. These findings are authenticated by the specific results, such as the good agreement of individual items within two raters and within two assessments; good internal consistency of Mini-BESTest’s items; and the excellent inter-and intra-rater reliability of the Mini-BESTest with the ICC. Further support for these findings is the result from the BA analysis, in which a good agreement was observed either between raters or between two assessments from the second rater. Moreover, according to the coefficient of determination, these findings are within the limits of agreement between two raters and that those between two assessments from the second rater are out of bias.

However, the SEM and MDC95 of the Mini-BESTest displayed a lower detectable change score to distinguish a noticeable change in balance when compared with other recent studies [33,39,40]. That may be due to the lower standard deviation of the results, which indicates that our findings show high similarity between the two rater results and between two assessments of the second rater.

Previous studies concerning the discrimination of the Mini-BESTest were used for the determination between fallers versus non-fallers, patients with or without postural response deficits, and low or high levels of functional ability in Parkinson’s disease and stroke patients. Nearly all recent studies also pointed out that the Mini-BESTest could predict the disease severity and provided better sensitivity and specificity in identifying individuals with an abnormal postural response and distinguishing fallers from non-faller [24,25,40]. In the present study, the Mini-BESTest allowed discrimination between participants who had been diagnosed as DPN using both subjective (questionnaires ≥ 4) and objective (physical examination ≥ 2.5) criteria versus those who were only diagnosed objectively (physical examination ≥ 2.5), whereas the long-form (BESTest) could not differentiate between both groups. This means that the shorter Mini-BESTest is better suited for discrimination than its long-form. However, as this study is limited to validating the discrimination between participants having and not having been diagnosed with DPN, this point may need to be confirmed in further studies.

In terms of the age groups in the present work, participants over 60 years old (*n* = 16 or 36.4%) showed slightly lower Mini-BESTest scores than those who were younger than 60 years. The difference score, in this case, was 1.06 with a 95% CI from 0.62 to 2.75, *p* = 0.2106; however, this difference was found not to be significant. Hence, this finding revealed that the age of patients (younger or older) may not have an effect on the scores of the Mini-BESTest in this study.

The present study followed the methodological procedure based on the well-defined protocol with standard instructions described in previous studies [21,30,33]. Furthermore, the use of two raters and assessments based on live observation instead of video recordings, which was deemed one of the strengths in a previous study [33], was also applied in the present work. While this may explain the excellent findings obtained, differences between the current and previous studies with regards to the type of disease, the age groups of participants, and examiners’ qualifications also need to be taken into consideration.

Notably, two sections of the BESTest, namely: (1) Biomechanical constraints and (2) Stability limits, were omitted in the abbreviated Mini-BESTest based on factor analysis due to the lack of contribution to the dominant trait (unidimensional construct of balance) [18]. However, almost all balance components, such as dynamic stability, transfers, gait, variation of support surfaces and visual conditions, obstacle negotiation, external forces, and performance during dual-tasking are incorporated in the Mini-BESTest assessment [18,41]. Hence, based on the excellent results discussed above, the validity and reliability of Mini-BESTest in type 2 diabetic peripheral neuropathy populations could be clearly demonstrated.

In our study, we chose to validate the discrimination of the Mini-BESTest instead of the concurrent validity. This was done since the Mini-BESTest is the short form of the BESTest, and we have already established the same correlation between the two in previous studies. Moreover, the Mini-BESTest shows better validation results than the BESTest in discrimination, although additional confirmation is needed in future studies. Another limitation in the current results is that the validity analysis is performed as only the discrimination validity and both the Mini-BESTest and BESTest were scored concurrently based on a single performance to reduce the participants’ fatigue or learning effects. Further study may be needed to confirm the concurrent validity by testing the correlation with other standard balance measures.

Moreover, this study used a narrow age range of 40–70 years regarding the type 2 diabetic patients with peripheral neuropathy selected for evaluation. Most participants in the current study were female; this is mainly due to the prevalence rate of diabetes being higher in females, but also as a result of female diabetic participants being more inclined to come to the hospital for treatment. Therefore, the inability to collect and match data for both sexes can be seen as a weakness of this study, which, in turn, means that the results may not be representative of the male population. To remedy these limitations, future studies should aim to examine the Mini-BESTest in age and sex matched groups. In addition, the clinical meaningfulness of the change score of the Mini-BESTest may need to be further explored by using standardized intervention methods.

## 5. Conclusions

The Mini-BESTest has been demonstrated to be a fast, simple, and non-invasive clinical tool with high reliability that fulfills requirements for stability and good distributions of its measurements regarding the balance performance assessment of type 2 diabetic patients with peripheral neuropathy. Moreover, the discrimination between a combination of subjective and objective examinations and solely objective examination for DPN diagnostics can be achieved by using the Mini-BESTest. Future studies are needed to explore the applicability of Mini-BESTest to discriminate between DPN with and without balance impairment and to correlate with the other standardized balance measures in order to expand the generalizability of the results.

## Figures and Tables

**Table 1:** Characteristics of the participants (*n* = 44).

Characteristics	*n* (%)	Mean ± SD
Gender, *n* (%)		
Male	4 (9.1)	
Female	40 (90.9)	
Age (years)		56.61 ± 7.70
≥60 years	16 (36.3)	
<60 years	28 (63.6)	
Marital Status		
Single	6 (13.6)	
Married	20 (45.5)	
Divorced	2 (4.5)	
Widowed	16 (36.4)	
Education, *n* (%)		
Primary	7 (15.9)	
Secondary	21 (47.7)	
Higher	13 (29.5)	
Graduate	3 (6.8)	
Occupation		
Unemployed	29 (65.9)	
Employed (e.g., government, business, worker, etc.)	15 (34.1)	
Weight (kg)		57.57 ± 10.68
Height (m)		1.56 ± 0.06
BMI (kg/m^2^)		23.77 ± 4.39
Smoking	3 (6.8)	
Alcohol drinking	3 (6.8)	
Duration of DM (years)		8.43 ± 3.30
Blood sugar level (mg/dL)		142 ± 39.52
Drugs controlling DM (how many tablets per time)		3.34 ± 0.99
HbA1C (mg/dL)		8.34 ± 2.01
MNSI Questionnaires; mean ± SD		4.16 ± 2.07
<4	17 (38.6)	
≥4	27 (61.4)	
MNSI Physical assessment		3.09 ± 0.66
Other underlying comorbidities (e.g., hypertension, heart disease, etc.)	37 (84.1)	
History of foot ulcer (no ulcer at present) *n* (%)	2 (4.5)	

DM, Diabetic Mellitus; BMI, Body mass index; MNSI, Michigan neuropathy screening instrument; SD, Standard deviation.

**Table 2:** Inter- and intra-rater agreement for a single item of the Mini-Balance Evaluation Systems Test (Mini-BESTest) and item-total correlation from *n* = 44.

Item	Single Item Agreement (Weighted Kappa)		Item-Total
	Inter-Rater	Test-Retest	
1	-	-	0.000
2	0.723	0.906	0.522
3	0.848	0.729	0.404
4	0.596	0.585	0.267
5	0.860	0.665	0.529
6	0.691	0.909	0.309
7	-	-	0.000
8	0.815	0.909	0.422
9	0.643	0.660	0.418
10	0.891	0.944	0.460
11	0.633	0.679	0.313
12	1.000	1.000	0.022
13	0.758	0.809	0.445
14	0.656	0.656	0.333

**Table 3:** Mean ± SD and intra and inter-rater reliability of Mini-BESTest from *n* = 44.

	Rater	Mini-BESTest Mean ± SD	Inter-Rater Reliability ICC_2,1_ (95% CI) *p*-Value	Intra-Rater Reliability ICC_3,1_ (95% CI) *p*-Value
First assessment	1	21.39 ± 2.71	0.95 (0.91–0.97) *p* < 0.001	0.93 (0.87–0.96) *p* < 0.001
	2	21.41 ± 2.50		
Second assessment	2	21.43 ± 2.43		
MDC_95_ *		2.16		

ICC, Intraclass correlation coefficient; ICC_2,1_, two-way random-effects; ICC_3,1_, two-way mixed effect; CI, Confidence interval; SD, Standard deviation; SEM. Standard error measurement; MDC, Minimal detectable change. (* Both SEM and MDC were calculated from the data of two assessments of the second rater).

**Table 4:** Comparison of the scores of the Mini-BESTest and the BESTest between the participants reporting MNSI’s subjective examination scores between ≥4 versus <4 (*n* = 44).

	Subjective Examination of the Michigan Neuropathy Screening Instrument (MNSI)		The Different (95% CI)	*p*-Value
	**<4 Score** **(*n* = 17)**	**≥4 Score** **(*n* = 27)**		
Mini-BESTest	22.65 ± 2.26	205.6 ± 2.65	2.09 (0.52 to 3.66)	0.0102
BESTest	91.47 ± 6.93	87.07 ± 8.12	4.40 (−0.41 to 9.20)	0.0718

MNSI, Michigan neuropathy screening instrument; CI, confidence interval; SD, standard deviation.

## Data Availability

The datasets generated and/or analyzed during the current study are available from the corresponding author on reasonable request.
